# Family caregiver experiences in terminal cancer: a qualitative study in palliative care

**DOI:** 10.1186/s12904-025-01965-2

**Published:** 2026-02-25

**Authors:** Masoud Rezaei, Tahereh Najafi Ghezeljeh, Ahmad Reza Baghernezhad, Reza Momen, Razieh Mohammadzadeh, Masoumeh Neishabouri, Naimeh Seyedfatemi

**Affiliations:** 1 Department of Medical and Surgical Nursing, School of Nursing, Arak University of Medical Sciences, Arak, Iran; 2 School of Nursing and Midwifery, Iran University of Medical Sciences, Tehran, Iran; 3 Cardiovascular Nursing Research Center, Rajaie Cardiovascular Medical and Research Center, University of Medical Sciences, Tehran, Iran; 4 Student Research Committee, Faculty of Nursing and Midwifery, Iran University of Medical Sciences, Tehran, Iran; 5 School of Nursing, Aja University of Medical Sciences, Tehran, Iran; 6 Department of Gerontological Nursing, School of Nursing, Abadan University of Medical Sciences, Abadan, Iran; 7 Nursing and Midwifery Care Research Center, School of Nursing and Midwifery, Iran University of Medical Sciences, Tehran, Iran; 8 Geriatric Mental health Research Center, Nursing & Midwifery Care Research Center, Health Management Research Institute, Iran University of Medical Sciences, Tehran, Iran

**Keywords:** Family caregiver, Caregiving experiences, Palliative care, Advanced cancer, Qualitative research

## Abstract

**Background:**

Caring for a family member with advanced cancer is a physically and emotionally demanding experience that can result in severe psychological distress. Understanding the challenges and burdens faced by family caregivers is essential to developing effective support strategies.

**Objective:**

This study aimed to explore the caregiving challenges and lived experiences of Iranian family caregivers of patients with advanced cancer.

**Methods:**

This qualitative study was conducted in Tehran, Iran, between March 2021 and April 2022. Semi-structured interviews were performed with 13 family caregivers, selected through purposive sampling with maximum variation, until data saturation was achieved. Data were analyzed using Graneheim and Lundman’s content analysis. Credibility and trustworthiness were ensured through member checking, peer debriefing, and adherence to Lincoln and Guba’s qualitative rigor criteria (credibility, dependability, confirmability, and transferability).

**Results:**

Participants, mostly women aged 35–75, described profound physical, emotional, and psychological burdens. The overarching theme, “Fence of Caring,” emerged, encompassing four categories: (1) physical and psychological collapse (2), being trapped in caregiving challenges (3), neglected caregiver, and (4) rumination on the death of a loved one.

**Conclusion:**

Iranian family caregivers experience multidimensional burdens within a sociocultural context that emphasizes familial responsibility. Recognition of these challenges is critical to designing culturally sensitive interventions that reduce caregiver strain and support preparedness for end-of-life care.

## Introduction

Cancer remains one of the leading health challenges worldwide, profoundly affecting not only patients but also their families [1]. In Iran, 131,191 new cancer cases and 79,136 deaths were reported in 2020, reflecting a mortality rate of 66.9 per 100,000 populations [2, 3]. Beyond the clinical implications, cancer disrupts the social and emotional fabric of families. In societies such as Iran where family bonds and collective responsibility are culturally embedded the diagnosis of cancer extends its impact to the entire household [4–6]. Iran’s predominantly Muslim and family-oriented culture encourages close emotional involvement in illness, often positioning relatives as the primary source of physical, emotional, and spiritual care [7]. Family caregivers are typically spouses, children, or other close relatives who provide unpaid, continuous support to patients facing chronic or life-threatening conditions [8, 9]. In advanced cancer care, the focus shifts from cure to comfort, encompassing symptom management, emotional well-being, and preparation for end-of-life stages [10]. These caregivers frequently experience anxiety, fatigue, and emotional distress as they balance caregiving duties with personal and family responsibilities [9–12]. Over time, this sustained burden may lead to depression, social isolation, and a decline in quality of life [13–15]. Global evidence consistently highlights the heavy psychological and social toll of caregiving. Studies from diverse regions show that caregivers of advanced cancer patients face unmet psychosocial needs, limited institutional support, and high emotional strain [16–18]. However, most existing studies have been conducted in Western or high-income contexts, where formal palliative systems are more established. The influence of cultural norms, family structure, and religious beliefs on caregiving practices in non-Western societies (particularly in Iran) remains insufficiently understood. Research indicates that the presence of structured palliative and supportive programs can reduce caregiver stress and improve family well-being [17, 19, 20]. Yet, within Iran’s family-centered context, the personal experiences, challenges, and coping strategies of caregivers remain largely unexplored. Understanding these perspectives is vital for designing interventions that are culturally sensitive and responsive to caregivers’ needs. Therefore, the present study aimed to explore the lived experiences and challenges of Iranian family caregivers of patients with advanced cancer, providing insight into their unique struggles within the sociocultural realities of caregiving in Iran.

Despite increasing research attention on the psychological and economic aspects of caregiving, little is known about how cultural and religious contexts shape caregivers’ interpretations of burden, resilience, and meaning in care. This study is significant because it explores caregiving within Iran’s unique socio-religious framework, where family obligation and faith strongly influence health behaviors and emotional coping. By addressing this cultural and contextual gap, the study adds an original perspective to the global understanding of caregiving in non-Western societies.

## Materials and methods

The present study was part of a larger mixed-method study [21] that aimed to elucidate the experiences of family caregivers with family-based dignity and expressive writing interventions. In the qualitative section of this mixed-methods study, participants not only shared their experiences of the interventions’ impact on anticipatory grief but also revealed insights into their overall caregiving experiences.

### Study design

This study employed a qualitative content analysis approach [22]. After obtaining informed consent for participation in the qualitative section of the study, semi-structured interviews were conducted with participants. Participants were asked to share their experiences of caregiving for a family member with cancer. Most interviews took place in a room at the Oncology and Hematology Department or the Alaa Palliative Room at Firuzgar Hospital in Tehran, Iran. However, some interviews were conducted in locations such as parks near participants’ homes, based on mutual agreement between the researcher and participant. Given the COVID-19 pandemic, strict preventive measures including wearing masks, maintaining physical distance, and regular hand hygiene were implemented during face-to-face interviews to reduce the risk of transmission. Additionally, seven interviews were conducted via telephone or virtual platforms such as WhatsApp or Skype when participants preferred remote communication. All in-person sessions were held only after obtaining permits from hospital authorities, and institutional safety protocols were strictly followed.

### Participants

In this study, participant selection was carried out purposively and continued gradually until the confirmation and completion of the main categories. The researcher examined the relationship between the data and asked further questions until data saturation was reached. In qualitative research, data saturation refers to the point at which additional data no longer yield new information or insights relevant to the emerging categories [22]. Although Graneheim and Lundman’s original framework for qualitative content analysis does not explicitly define the notion of saturation, many scholars have integrated it as a complementary indicator of analytical completeness in content analysis studies [23, 24]. In the present study, saturation was conceptualized as the stage when no new meaning units, codes, or categories emerged from subsequent interviews, ensuring adequate coverage and representativeness of participants’ lived experiences. This operationalization aligns with the iterative and inductive nature of Graneheim and Lundman’s approach, which emphasizes repeated review, comparison, and abstraction of data to achieve comprehensive understanding [25]. Based on this principle, participant recruitment continued until data saturation was achieved, which occurred after the twelfth interview. In total, thirteen family caregivers of patients with advanced cancer were interviewed. To ensure a diverse range of experiences and perspectives a maximum variation sample was recruited, considering factors such as gender, age, education level, relationship, and cancer type. The characteristics of the participants are presented in Table 1.

**Table 1:** Characteristics of the family caregivers and their family member

Caregiver	Gender	Age, y	Education Status	Relationship	Interview time (min)	Interview Method
CG1	Female	46	Diploma	Wife	68	Face-to-face
CG2:	Female	38	Postgrad	Mother	40	Virtual
CG3:	Male	48	Undergrad	Son	45	Virtual
CG4:	Female	40	Diploma	Mother	35	Virtual
CG5:	Female	38	Diploma	Daughter	75	Face-to-face
CG6:	Male	62	Diploma	Father	36	Virtual
CG7:	Male	34	Diploma	Daughter	55	Virtual
CG8:	Female	46	Diploma	Daughter	49	Face-to-face
CG9:	Male	34	Postgrad	Son	61	Face-to-face
CG10:	Female	40	Undergrad	Daughter	47	Virtual
CG11:	Female	34	Diploma	Mother	44	Virtual
CG12:	Female	32	Diploma	Daughter	49	Face-to-face
CG13:	Female	39	Diploma	Daughter	74	Face-to-face

Family member	Age,y	Gender	Education Status	Type of Cancer	Job Status	
FM1:	52	Male	Diploma	Liver	Clerk	
FM2	14	Female	Student	Brain	Student	
FM3:	73	Male	Primary Education	Stomach	Retired	
FM4:	13	Male	Student	Sarcoma	Student	
FM5:	68	Male	Diploma	Lymphoma	Retired	
FM6:	23	Male	Undergrad	Lunge	Unemployed	
FM7:	26	Female	Diploma	Esophagus	House keeper	
FM8:	73	Female	Primary Education	Colorectal	House keeper	
FM9:	75	Male	Primary Education	Colon	Retired	
FM10:	69	Male	Primary Education	Colorectal	Retired	
FM11:	17	Female	Student	Sarcoma	Student	
FM12:	59	Female	Diploma	Colorectal	House keeper	
FM13:	79	Male	Primary Education	Bladder	Retired	

### Data collection

In the original project, semi-structured interviews were developed based on the literature on family-based dignity intervention and expressive writing [26, 27]. The primary aim of these interviews was to explore participants’ perceptions of the interventions they had received. However, during the course of the interviews, participants spontaneously and extensively reflected on their broader caregiving experiences, including physical, emotional, and social challenges they faced while looking after their loved ones with advanced cancer. Before each interview, the researcher established communication with the intended participant via telephone call, providing necessary explanations about the interview’s objectives and the participant’s valuable role in the research. With the participant’s consent, arrangements were made regarding the time and place of the interview. At the beginning of each interview session, the researcher introduced themselves, explained the purpose of the interview, and assured participants of the confidentiality of their information before obtaining audio recording permission. To ensure consistent data collection, semi-structured interviews were conducted using an interview guide that was developed based on the objectives of the study and relevant literature on family-based dignity intervention and expressive writing. The guide included two types of questions: (a) core questions, which explored participants’ experiences and perceptions of the interventions they received, and (b) follow-up probing questions, which encouraged elaboration and clarification (e.g., “What do you mean by…?”; “Can you give an example?”). Thes questions were used to delve deeper into participants’ perceptions and experiences [24]. Examples of the core questions included:“Please describe your understanding and experience regarding the family-based dignity intervention sessions or the expressive writing assignments.”, “How did reviewing the written transcripts of your sessions affect your perception and caregiving experience?”,“After reflecting on memorable memories discussed during the sessions, how did this influence your relationship and caregiving for your family member?”,“Please tell us about the events or experiences that occurred after the intervention ended.”For the purposes of the present article, we did not analyze participants’ feedback on the interventions themselves. Instead, we conducted a focused analysis of the narratives that emerged regarding the caregiving trajectory. In other words, while the interview guide was originally constructed around intervention-related questions, the rich qualitative data provided deeper insights into family caregivers’ lived experiences, which became the analytical focus of this study.

The researcher initiated the interview while minimizing interference to encourage participants to freely express their views and experiences. Prior to the interview, participants were informed of the approximate duration to accommodate their schedules, although the actual interview length varied. Each interview lasted between 35 and 75 min. Both face-to-face and virtual interviews were audio-recorded and stored on a password-protected home computer, with each interview assigned a unique code. Data collection continued until data saturation was reached, which occurred after the twelfth interview. In total, thirteen family caregivers participated in the study.Data collection occurred between February 2021 and March 2022.

###  Data analysis

In this study, Graneheim and Lundman’s conventional content analysis was employed to explore participants’ perceptions and experiences [22]. Immediately after each interview, the audio recordings were transcribed verbatim. All transcripts were then coded and managed using MAXQDA 20 software. Each interview was analyzed individually, with the text reviewed multiple times to familiarize the researcher with the data and gain a general understanding. Semantic units—words, sentences, or paragraphs containing significant information about participants’ experiences—were identified and labeled with summary codes. Codes were refined iteratively, merging similar ones and classifying them into categories. To ensure rigor, coding and categorization were not conducted in isolation. After the first author (MR) completed initial coding, two researchers (TNGH, NSF) with backgrounds in qualitative research and nursing, including palliative and critical care, independently reviewed selected transcripts and the preliminary coding framework. Differences were discussed until consensus was reached, and the coding scheme was adjusted accordingly. Regular team meetings were held to review emerging categories and themes, ensuring interpretations remained grounded in the data. This peer review and iterative discussion enhanced the credibility, dependability, and confirmability of the analysis. Through careful reflection and comparison of categories, the underlying meanings were distilled into themes. In line with accepted qualitative standards, the research team monitored data saturation to confirm analytical sufficiency and category stability, consistent with recommendations by Saunders et al. (2018) and Hsieh and Shannon (2005). This approach ensured both methodological transparency and credibility in applying content analysis to lived-experience data.

#### Trustworthiness

Lincoln and Guba (1985) proposed four criteria for evaluating the trustworthiness of qualitative research: credibility, dependability, confirmability, and transferability [28]. To ensure credibility, participants were selected from a diverse pool of family caregivers, and long-term engagement with sufficient time for data collection and analysis was allocated to deepen and contextualize the data. For dependability, the methodology section detailed the operational stages, and initial codes along with examples of theme extraction and excerpts were presented for external scrutiny by two independent qualitative researchers with expertise in oncology and palliative care. They reviewed the coding framework, themes, and selected transcripts, and provided feedback that was incorporated into the final analysis.To enhance confirmability, data was extracted directly from participants’ conversations, and researchers maintained objectivity by setting aside their personal views and motivations. Documenting all research stages and seeking expert opinions further strengthened data confirmability. To promote transferability, the study provided a comprehensive explanation of all details, including demographic characteristics, study context, participant selection, data collection methods, and analysis process. Findings were presented in detail with relevant quotations, and the study’s conduct and activities were described in a manner that facilitated reproducibility.

#### Ethical considerations

This study was approved by the Research Ethics Committee of Iran University of Medical Sciences (Ethical code: IR.IUMS.REC.1399.1097) and was conducted in accordance with the principles of the Declaration of Helsinki. Prior to data collection, the researcher introduced themselves to each participant, explained the study’s objectives, and answered any questions. Written informed consent was obtained from all participants, who were assured of complete confidentiality and informed of their right to withdraw at any time without penalty. Participants also granted permission for audio recording of their interviews, solely for research purposes. Preventive health considerations during the COVID-19 pandemic, including minimizing unnecessary contact and providing personal protective equipment, were also integrated into the study’s ethical framework.

## Results

**Fig. 1:**
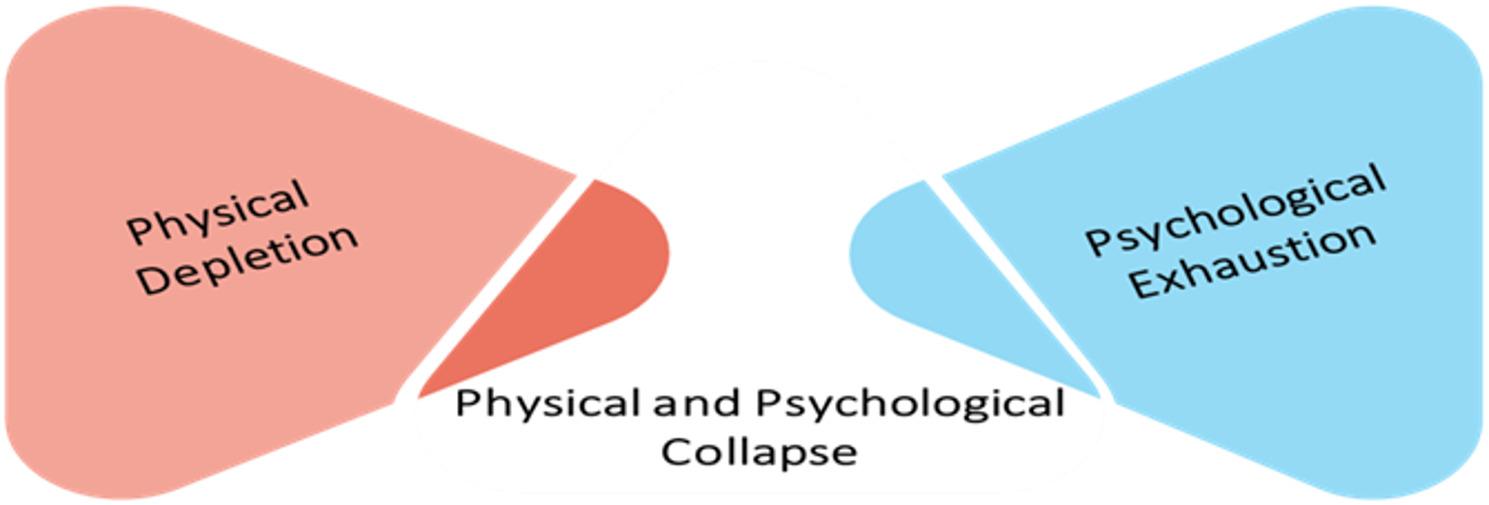
The first category of fence of caring

**Fig. 2:**
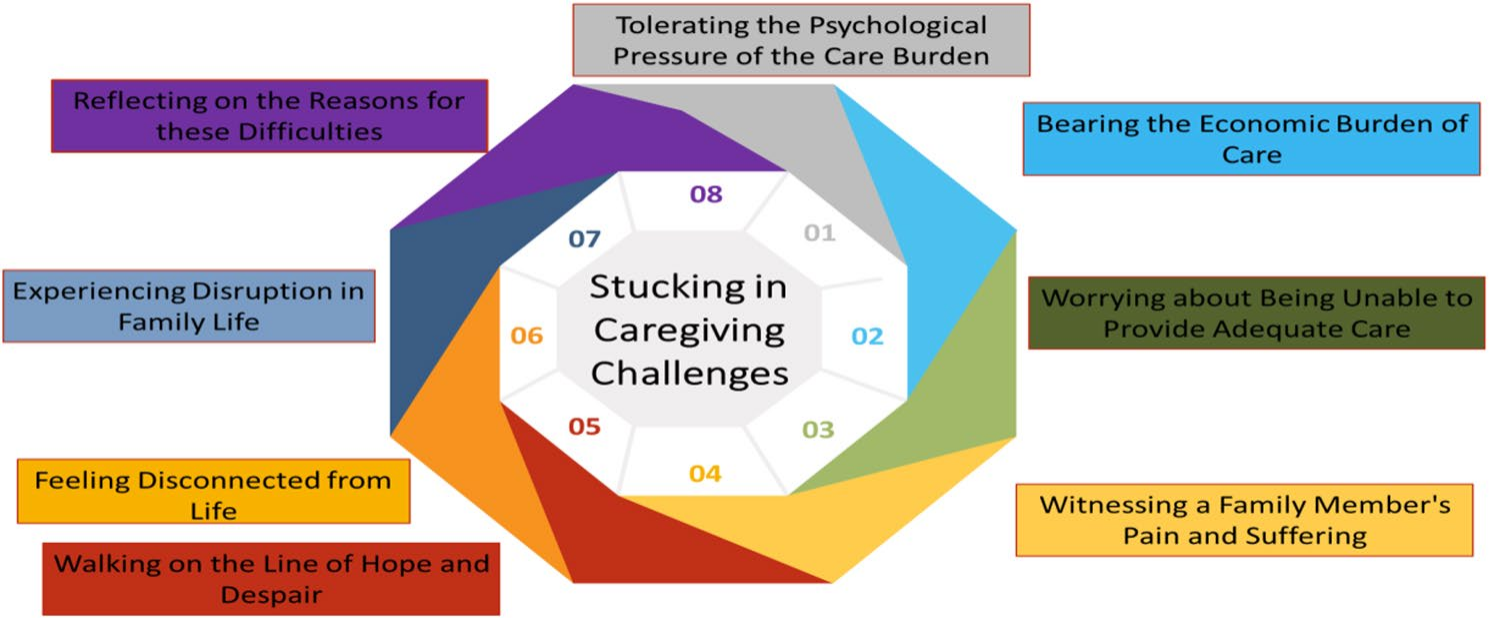
The second category of the fence of caring

**Fig. 3:**
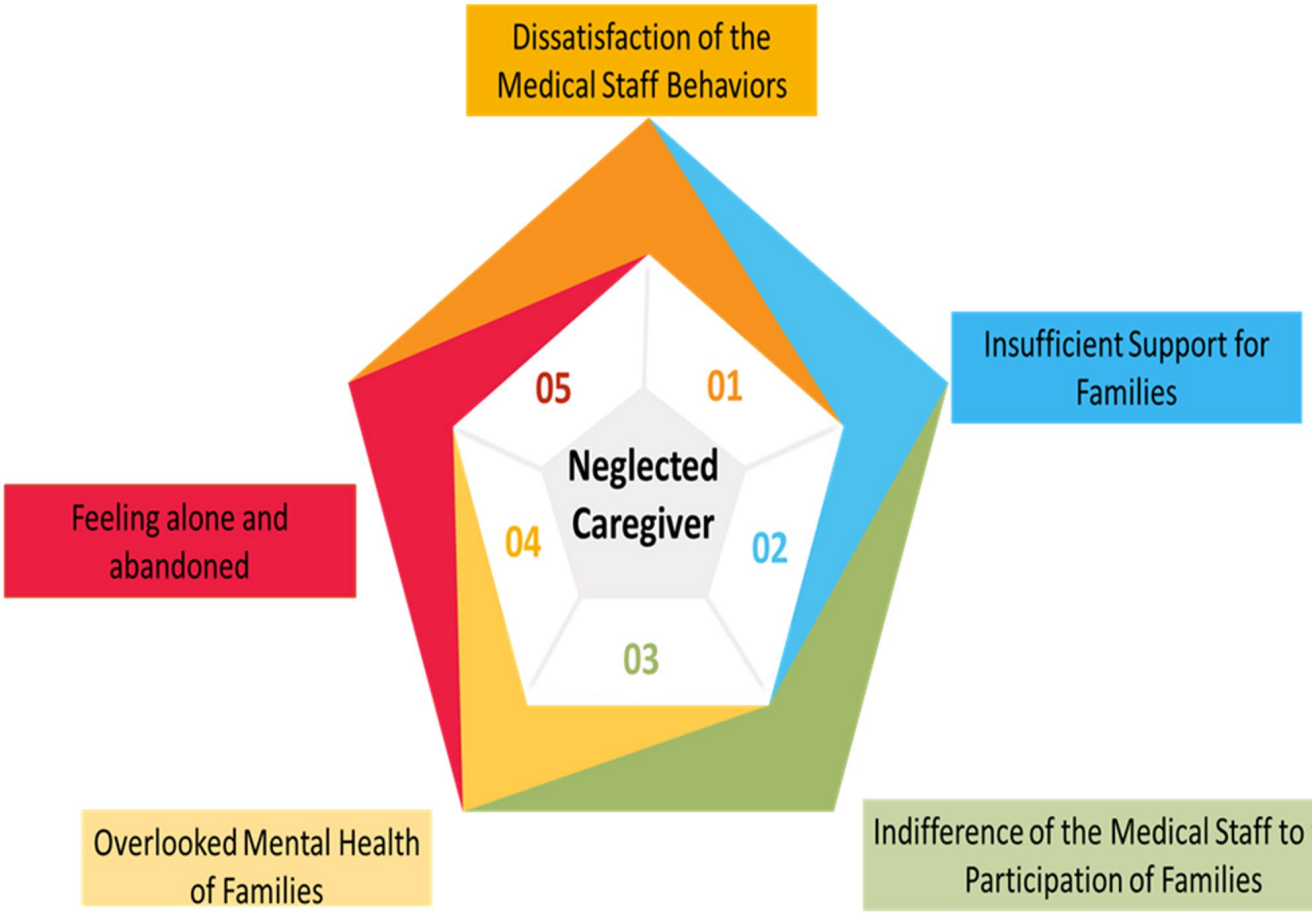
The third category of the fence of caring

**Fig. 4:**
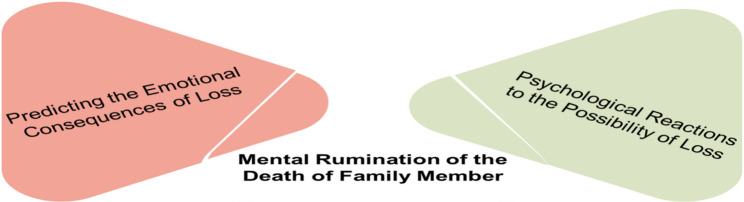
The fourth category of the fence of caring

The majority of participants were women aged 35–75 years, mostly caring for parents with advanced cancer. Data analysis led to the emergence of the overarching theme “Fence of Caring”, encompassing 17 subcategories within four main categories: (1) physical and psychological collapse (2), being stuck in caregiving challenges (3), neglected caregiver, and (4) mental rumination on the death of a loved one (Table 2; Figs. 1, 2, 3 and 4).

**Table 2:** Fence of Caring: Categories, sub-categories, and some quotes of the participants

Theme	Category	Sub-Category	Participants’ statements
Fence of Caring	Physical and Psychological Destruction	Dyeing Down of Physical Strength	One caregiver, tending to his wife with advanced liver cancer, shared his thoughts on body weight analysis: *“…Dealing with a disease like my wife’s is like placing something precious at the top of a mountain I’m striving to reach. I disregard the difficulty of the path and focus solely on reaching it sooner. It’s as if the object has been moved further or higher. I try not to be daunted by the challenges of the journey*,* but they actually fuel me. It’s exhausting. I feel physically and mentally drained by these obstacles on the path*,* but striving for that dream or reaching the peak of the mountain gives me motivation. I’m just a few steps away from getting there. Yet*,* it feels like I’m being pushed further back. Since my wife’s illness began*,* the further I go*,* the more I encounter closed doors.” (P1)* Arguably, one of the most poignant insights from the physical examination findings came from a son whose father was in the advanced stages of stomach cancer: *“…The presence of such issues for one family member naturally impacts other family members. In economics*,* it’s akin to the spillover effect*,* like how a natural disaster such as an earthquake in one country can affect the economies of other countries.* The impact of this illness on the patient is so significant that it spills over and involves others as well. The patient suffers until morning, and as a caregiver, I won’t rest until morning either. Unconsciously, the patient’s problems also affect my health. It takes a toll on your body and soul. You become vulnerable. Your energy depletes” (P3).
		Psychological Exhaustion	In line with this finding, the participants stated that they experienced emotional and psychological fluctuations while caring for a family member: *“…I realized how much mood swings I had in this one year… For example*,* once I became hopeful*,* I sat down to dhikr*,* to pray*,* to pray*,* to pray*,* and these things*,* then all of a sudden*,* all my works were destroyed and I went back… well*,* I really have to… I mean*,* I am not innocent to be able to work on myself so that these things don’t affect me… Now I am in a bad mood for a few days*,* I am desperate for a few days so that I can be with him again later. The things that I learned*,* to rebuild myself… well*,* these mental and emotional fluctuations generally take a person to the phase of depression*,* nervousness*,* lack of moral balance at all… which maybe the person himself does not realize” (P1).* In relation to the finding of mental exhaustion, other participants stated that they experienced depression and impaired concentration. *“…My heart is in chaos*,* I keep saying*,* God*,* I wish I was in my daughter’s place right now on the bed. Because life is no different between me*,* who is healthy*,* and my daughter*,* who is on the bed*,* we are both suffering. The only difference between my pain and my daughter’s is the pain. My daughter is getting better with painkillers*,* but there is no cure for my mental pain… My mental condition is getting worse day by day… I don’t feel tired*,* I just feel depressed… How many times a day do I sit and think about you? … while I’m thinking*,* I’m doing something that I shouldn’t have done at all… Sometimes I walk around the house and forget what I wanted to do…” (P11).*
	Stucking in the Buttelencks of Care	Tolerating the Pressure of Caring	Consistent with this discovery, participants reported experiencing emotional and psychological fluctuations while caring for a family member: *“…I realized how much my mood swung in this past year… For instance*,* when I felt hopeful*,* I would engage in dhikr*,* prayer*,* and meditation*,* and then suddenly*,* everything would fall apart and I’d regress… I really need to… I mean*,* I’m not blameless*,* I need to work on myself so these things don’t affect me… Now I’m in a bad mood for a few days*,* then I’m desperate for a few days just so I can be there for him later. The things I’ve learned to rebuild myself… well*,* these mental and emotional fluctuations often lead a person to a state of depression*,* anxiety*,* and a lack of emotional stability… which the person may not even realize” (P1).* Regarding the issue of mental fatigue, other participants expressed experiencing depression and difficulty concentrating. *“…My heart is in turmoil*,* I keep thinking*,* God*,* I wish I were the one in bed right now instead of my daughter. Because there’s no difference in suffering between me*,* who is healthy*,* and my daughter*,* who is in bed. The only difference is the physical pain. My daughter’s pain can be alleviated with painkillers*,* but there’s no cure for my mental anguish… My mental state is deteriorating day by day… I don’t feel physically exhausted*,* I just feel depressed… How many times a day do I sit and think of you? … while I’m thinking*,* I end up doing things I shouldn’t be doing at all… Sometimes I wander around the house and forget what I intended to do…” (P11).* In line with the challenges of caregiving, one participant mentioned spending a considerable amount of time meeting the basic needs of their child, thus neglecting their own essential needs. *“…there were difficult days*,* I was alone with him*,* we had no one to assist us*,* to open a door for us… Financial constraints*,* sleepless nights*,* hunger in this city*,* we couldn’t find medication*,* I was in the hospital for a while*,* dealing with COVID… I was afraid to buy anything from outside in fear of infecting my child*,* I didn’t even dare to use the restroom here*,* in case my daughter caught COVID if it was present… I mean*,* every moment*,* whatever I do at home*,* I have to check on him and see if he needs anything*,* if he’s okay. Every moment he calls me*,* mom*,* come here*,* mom*,* let’s go out*,* mom*,* take me to the bathroom… Mom*,* come wash my hands… give me the toy… I say Saddam a hundred times. Let mom come and do this… I have two other children*,* my husband is there*,* it’s really hard…” (P11).* Another participant mentioned that not only the patient, but the family members are also suffering, and their living conditions are entangled in the complexities of caregiving issues. *“…unusual things happened to us… bitter experiences that we all shared due to mom’s illness… it’s not just mom who is suffering*,* I*,* my brother*,* and my father are also struggling. We are in pain… Our lives have taken a turn and have become very challenging for us… from one hospital to another*,* from this pharmacy to that pharmacy… our life plan has been disrupted… believe me*,* being the companion of an incurable patient is torturous step by step. It’s excruciating… it requires attention… here we are*,* the nurses say*,* go with so-and-so and get this medicine*,* come… I don’t know how to leave my patient alone*,* I’m going to look for medicine… what’s happening? Provide facilities in this hospital so that at least the patient’s companion doesn’t have to search for medication…” (P12).*
		Thinking aboute the Reason of Difficalities	Participants discussed the challenges of self-reflection and the impact of others’ perceptions on their experiences. One participant questioned whether others see their suffering as a result of personal failings or external circumstances. They emphasized the importance of the psychological dimension in enduring hardship and pondered the basis on which they endure such challenges. The participant expressed a desire to understand how others perceive them and the reasons behind their endurance.
		Tolerating the Mental Pressure of Caring	*Perhaps our current multitude of problems stems from our unwavering focus on the party’s inability to emerge from the depths. Ultimately*,* we yearn for a return to normalcy*,* yet these families are ensnared in the grip of this affliction. They endure immense psychological strain*,* tarnishing the sweetness of days gone by. (P3).* *…I’ve been grappling with a skin issue. Upon visiting the dermatologist*,* I was informed that it stems from my mental unrest. My mind is in disarray. Often*,* I keep my thoughts to myself*,* avoiding burdening my mother. Witnessing her tears is incredibly taxing*,* and I find myself on the verge of tears constantly. It’s an arduous struggle for me. (P12).*
		Bearing the Economic Burden of Care	The contributors express their economic concerns: “…*The biggest thing that has bothered me during this period*,* especially in the next one or two months*,* is economic… For example*,* I am paying installments*,* I am paying Snap fees because the hospital is in your plan… It is really a question for me that now You think that you are checking from a psychological point of view… because when there is a financial problem*,* you don’t talk about feelings anymore… for example*,* just now my landlord told me that you should get up… well*,* I think since two months My wife is at the peak of her illness*,* and I’m already thinking… Where should I go to stay… With this money in advance and rent*,* I have nowhere to go… Well*,* these financial problems are very annoying… Well*,* you are on your own. You know how special chemotherapy patients have to be in terms of nutrition. You may not believe that some months I can’t even eat a kilo of meat… Then I have to give my wife quail*,* fish*,* in this situation that I eat once every 6* months I don’t buy fish myself… even *if I buy a juice*,* well*,* this is very annoying… this economic discussion is one side… and well*,* I am very sensitive not to ask anyone for help…” (P1).* *“…everything my daughter wants should be provided… that is*,* if she says she wants gold*,* we have to get it for her… she doesn’t think about all this trouble and hardship*,* house rent and all these expenses. When he says that he wants such and such clothes*,* I want such and such shoes*,* we have to get them… We also have to*,* if we don’t get them*,* he will make a fuss about him… Every cancer patient is not only his disease*,* he really has expenses*,* it’s very difficult… in a bad house They complain…“(P11).*
		Worrying not being able to care	One participant expressed concern about the responsibility of caregiving, saying *…"I was alone at that time and I thought*,* if something bad happens to my father*,* what can I do? After all*,* you accept responsibility and you are helping someone who is the elder of the family. You are also stressed that if it doesn’t work*,* And he did it… I myself am one of the people who feel too responsible and self-absorbed… In my notes*,* I kept mentioning that if an accident happens now*,* God forbid*,* what should I do*,* and I wrote myself*,* I wrote my worries… it’s all anxiety*,* stress*,* the expectation of the other party*,* and in a word*,* it’s like a sick person takes refuge in you…” (P3).* Another contributor attributed this concern to the experience of being alone in caregiving, stating, *“…because in this condition of mom’s illness*,* everyone is thinking of emptying their shoulders and leaving you… maybe not everyone would be satisfied with mom’s moans*,* even if she was at home… my brother used to go to his friend’s house Don’t listen to mom’s moans… Well*,* in this situation where there is no one alone*,* you are afraid that you won’t be able to take care of your sick… I often fear that mom’s condition will become such that I can’t handle it anymore…” (P12).*
		Difficulty Seeing the Pain and Suffering of a Family Member	One participant described his father’s physical condition as follows: *“I always saw my father as a strong man with strength*,* health*,* and cheerfulness in my mind. Now I see that an illness has caused him to be bedridden and unable to walk. He has lost weight*,* we have nothing left in his body… this makes you sad…” (P9).* Another participant described seeing the pain of a family member as a kind of mental torment or a sad experience: *“I feel sad when I see that life is ruined for me… I feel very sad when I come to this environment… I am sad since I opened my eyes this morning and when my eyes fall on the sick rooms*,* I feel emotional torment…” (P12).*
		Walking on the Line of Hope and Despair	In relation to this discovery, the participants shared their experiences in the following manner: *“…I’m completely out of my mind. I want to explain where I’ve been*,* which doctors I’ve visited*,* and the hope they’ve given me. I lived with hope for a few days*,* as I was supposed to*,* and I finally reached* the place I was meant *to reach*,* my ultimate destination. My wife’s illness and my children’s confusion have troubled me several times. They ask*,* what do the doctors say? What do they understand? Then I hope that perhaps God wants to close all the doors that are about to open*,* so I must open them. I’m moving forward solely with this hope…” (P1).* *“Now*,* sometimes when I return home and see my mother*,* I look into her eyes. I thank God that I get to see her for another day… Sometimes I feel very hopeful*,* and I wish for more time with my mother. At times*,* despair overwhelms me… when I pray for God to protect my mother and when I fall into despair*,* I express my gratitude to God…” (P8)*
		Presence of Disorder of Family Life	One participant expressed that looking after his father has led to him spending less time with his wife: *“…With all the troubles and pressure*,* I feel a sense of depression. I find myself consumed with my father*,* and as a result*,* I have hardly seen my wife twice in the past few months” (P13).* Another participant mentioned that taking care of a family member has caused her to lag behind in her life, and her daily routine revolves around her mother’s health: *“…We’ve been here for almost a month*,* feeling exhausted sooner than expected. Both physically and mentally*,* we’re struggling. We’re not able to be with our children and wife*,* as they are managing on their own*,* but it’s difficult to miss out on certain aspects of life. It feels as though our lives revolve around our mother’s condition. When she’s home from the hospital*,* we feel relieved*,* but when she’s there*,* time seems to stand still for us. It’s hard for things to progress…” (P8).*
		Staying Away from Life	One participant expressed that when a family member fell ill, it felt like the world came to a standstill for them: *“…but right now*,* as I speak to you*,* it’s like the world has come to a halt… I want to start exercising*,* but I think of my mother… or when I move around the house*,* something inside me tells me to stop. It’s as if someone is guiding my hands*,* urging me not to proceed…” (P8).* Other participants highlighted how caregiving caused disruption and disorder in their lives: *“…it makes you feel really awful… and we can’t tend to our personal matters… my sister and I were attending language classes*,* going to the gym*,* but we had to cancel everything… we signed up but couldn’t find time to study… and this disruption affects not just me*,* but the rest of the family members as well…” (P13).*
	Family Caregiver as a Left of Attention	Dissatisfaction of the Medical Staff Behaviors	In confirmation of such a finding, one of the participants admitted that he himself witnessed the misbehavior of the personnel with one of the caregivers: *“…this sentence shook me so much… you can’t believe it… it shook me so much… Ah… he returned to his mother and said that I didn’t give you this child… Go see what you did that God placed such a child in your lap I mean*,* you might not believe me*,* I have nothing to do with that mother*,* I was crushed there… but I was crushed… I mean*,* the society is the same… the behavior of the treatment personnel should be the way of work…” (P2).* Also, another participant describes his dissatisfaction with the treatment team as follows: *“… I would like to interview you… but one thing that annoys me is your colleagues… they don’t behave well with patients… well*,* I know their job is hard… two nurses with 30 Until the patient finally finds something wrong from somewhere*,* but anyway*,* the person who works in this department accepts all the problems… That day*,* I tell the nurse that my mother’s head is over and she wants to go to the service… if possible Come on*,* take it apart… He says*,* “What’s wrong with my head?! Don’t you know?! Change it yourself…” (P8).*
		Lack of Family Support Resources	In this regard, one of the participants stated that there was no financial support from government agencies or authorities, and they survived these conditions with the wife’s disability benefits: *“…no support from the government… from the organization… even the insurance pays two thirds of our salaries… think of an employee with 6200 salary and benefits and all overtime…*,* for example 1200 pieces It’s possible because of overtime and I’m paying 5 tomans in installments… Why doesn’t this society think about it? Now a salary of 5 tomans that has insurance pays two-thirds of it is 3700… Well*,* how can I plan?.“(P1).* Also, in confirmation of this finding, another participant says the following about the lack of psychological support resources: *“…one of my wishes as having a special child is that… are children really abandoned like that in other countries? And a child who has suffered so many physical and mental injuries*,* you assume my daughter He was supposed to go to school*,* he wet himself… I took him to school after an hour and told him to come pick him up… Lose half of your hair at the age of 8?… I really want these children to be psychologically supported..psychiatry is something for these children… there is no support at all for families with special children…” (P2****)***
		Indifference of the Medical Staff to the Participation of Families	The statements of the participants confirm this finding: *“… I also told you that one day one has to leave this world*,* that we more or less accepted the mother’s conditions… Give us some advice so that we don’t come to ask ten times… We will be a nuisance to the nurse as well as a nuisance. We will get better or bother the doctor’s assistant… Once they explain everything to us*,* we take it and sit down… It’s as if a dropper is giving us the answer… It’s been a year since my mother’s illness and I’m still recovering from it. We don’t know what’s going on… they don’t care about our opinion at all… they don’t ask us for an opinion in any field… they only say once in a while that you should agree… you don’t dare to give an opinion at all… As long as you say something*,* they say take your patient…” (P8).* *“…I say*,* let me make a room here for myself and do some service… But I know that I will hit the wall when I go… It doesn’t fit. Yes*,* I was thinking a lot… For example*,* what do I know? I will go to the hospital. And fill out a form with the patient’s companions… go talk to them*,* for example… at least participate in the treatment of my patient… but no one has asked us for an opinion so far…” (P2).*
		Neglect of the Psychological Conditions of Families	*“…Now I don’t know*,* but I haven’t seen anything in these few years… I haven’t seen anything that even one person came to say what is your mental state?!! You have been suffering all this time and you have suffered*,* now your soul and spirit How is it?!…” (P2).* *“…they are not to blame either… but I think that the person who is working here has accepted the problems of this job and must have been fully trained… The people who work in this department should take a psychology course Let them see… I also told you about this series that we kept my mother for a year*,* that is*,* we were not bothered as much as we were here for a month… They bothered us a lot… One of the things that bothers us a lot is that the result They don’t tell us the right things… They don’t give us the right answers… We are hurt mentally and emotionally… It’s as if we*,* the comrades*,* are being held back… No one pays attention to us…” (P8*
		Feelings of Alone and Loneliness	One of the participants in this regard states as follows: *“…After a while I felt alone… there is no one around… It was as if I was in a dark place and a candle was in my hand and a strong wind was blowing and I wrapped my arms around the candle and the wind and the storm went out. Don’t do it… But instead*,* it’s like a fire is burning my own hand… I felt like taking care of my mother and it was hard for me to be alone…” (P7).* Also, other participants have experienced the feeling of loneliness and no one from relatives and acquaintances and stated that they did not have any kind of support from friends and acquaintances during the family member’s illness. *“…while writing*,* I remember the days when we were alone in Tehran… there was really no one to help me*,* no one… I was the best sister for my brother in his bad days… I would solve any problem I had.. but this happened to us*,* none of them asked at least once where are you? What do you have? What are you doing? Who is helping you? Where do you get the medicines? How do you pay for these operations?. I didn’t have anyone… Or my father-in-law really didn’t do anything for us… In this illness*,* I don’t know why they all rejected us…” (P11).*
	Mental Rumination of Death of Loved one	Psychological Reactions to the Possibility of Loss	In this regard, the participants state as follows: *“… My life is putting a lot of pressure on me right now… I mean*,* not thinking about my wife makes me hot… You can’t believe it*,* I mean I even pray in the middle of the night not to wake up… because when I wake up*,* not thinking about my wife … For example*,* on the days when he is in the hospital*,* my crying increases tenfold… I mean*,* I can’t even bear to be away from him for a few hours… Now*,* for example*,* the thought of not having this at all is very annoying…” (P1).* *“…in general*,* if you want to look at it from a psychological point of view*,* neither the past is important*,* nor the future*,* we have to find out right now… but the past is already rejected*,* so I don’t want to get too involved in it… but the future is because I think that my mother is no longer in my mood… and for a moment I am in the kitchen washing dishes*,* and if one day my mother is not there*,* it seems that the world will collapse on me…” (P8).*
		Predicting the Emotional Consequences of Loss	Below are some examples of the statements of the participants who say that in the absence of a father or mother, there is a possibility of disintegration of the unity and solidarity of family members. *“…for a series of families who are really good together and this feeling is strong between them*,* it is very difficult to feel sorry for each other and to think about separation… I mean*,* I really knew a teenager who died because of the death of his grandmother. He hanged himself… This issue is really so heavy that you don’t even want to think about it and you want to be alone and your loved ones leave you… and I see the day when there is no more father*,* what will happen to us children. …” (P9).* Another participant feels that he will lose a powerful supporter in life with the possibility of losing a family member: *“Reviewing memories may make you a little sad… because we finally had sweet moments with your father*,* you feel a little strange when reviewing memories… you feel that maybe you are going to know the path of life from now on. Supporter*,* you should move forward… and that nostalgia is not going to happen again… or those happy moments we had with our father and that it is not going to happen again*,* maybe it will make you feel a little regretful… it will also bring a little sadness. …” (P3).*

A) Physical and Psychological Collapse.

This category included two subcategories physical depletion and psychological exhaustion reflecting the toll caregiving takes on both body and mind (Fig. 1).

Physical Depletion: Caregivers described extreme fatigue and loss of energy.

*“As my father suffers through the night*,* I cannot rest… the patient’s pain drains my body and soul” (P3).*

Psychological Exhaustion: Emotional strain manifested as depression, sadness, and lack of focus.

*“My mental state worsens daily… I feel depressed and often wander without purpose” (P4).*

- B) Being Stuck in Caregiving Challenges.

This category reflected multiple caregiving burdens, structured into eight subcategories (Fig. 2).

Psychological Pressure: Caregivers hid their own distress to avoid upsetting patients.

*“I keep my thoughts to myself… witnessing my mother’s tears is unbearable” (P12).*

Economic Burden: Financial costs compounded stress.

*“The greatest challenge has been financial—rent*,* installments*,* and treatment expenses” (P1).*

Worrying about Adequate Care: Fear of failing to meet patients’ needs was common.

*“I worry my mother’s condition may deteriorate beyond my ability to cope” (P12).*

Witnessing Suffering: Observing loved ones’ decline was heartbreaking.

*“I always saw my father as strong… now he has wasted away*,* and it breaks my heart” (P9).*

Hope and Despair: Caregivers alternated between optimism and hopelessness.

*“Sometimes I feel hopeful*,* other times despair overwhelms me” (P8).*

Disconnected from Life: Other aspects of life were neglected.

*“Our lives revolve entirely around my mother’s condition” (P8).*

Family Disruption: Daily routines and responsibilities were interrupted.

*“It feels like the world has stopped… I cannot focus on anything else” (P8).*

Reflecting on Reasons: Caregivers sought meaning in their suffering.

*“Why did this have to happen to my father? Why must we endure this?” (P10).*

- C) Neglected Caregiver.

This category captured caregivers’ sense of being overlooked by health systems and social networks (Fig. 3).

Dissatisfaction with Medical Staff: Some felt disrespected by care providers.

*“I asked for help adjusting my mother’s scarf*,* and the nurse replied harshly*,* ‘Can’t you do it yourself?’” (P8).*

Insufficient Support: Families highlighted lack of financial or institutional aid.

*“There’s no support from the government or insurance… it barely covers our expenses” (P1).*

Indifference to Family Involvement: Caregivers were excluded from decisions.

*“They never ask for our opinion… we feel invisible in the process” (P8).*

Overlooked Mental Health: Emotional well-being of caregivers was neglected.

*“All these years*,* no one has asked about my mental state” (P2).*

Feeling Alone: Caregivers reported isolation and abandonment.

*“It was as if I were in a dark place holding a candle in a strong wind… bearing the burden alone” (P7).*

- D) Mental Rumination on the Death of a Loved One.

This category highlighted caregivers’ continuous preoccupation with the possible loss of their family member (Fig. 4).

Psychological Reactions to Loss: Anticipation of death caused severe emotional distress.

*“The thought of losing her is devastating… I cannot bear it” (P1).*

Predicting Emotional Consequences: Caregivers imagined the family’s future grief.

*“I wonder what will become of us children when our father is gone” (P9).*

## Discussion

In this study, the experiences of Iranian family caregivers caring for loved ones with advanced cancer were explored through the overarching theme of the “Fence of Caring.” This theme encapsulates the multifaceted and interrelated physical, emotional, social, and existential burdens faced by caregivers. Unlike studies conducted in Western contexts, where formal palliative systems and professional supports are more established, caregiving in Iran unfolds within a sociocultural framework that places strong moral and religious emphasis on familial responsibility and endurance. Within this cultural setting, caregiving becomes both a moral duty and a spiritual test, shaping caregivers’ interpretation of suffering, sacrifice, and resilience.

The first category, Physical and Psychological Collapse, reflects the chronic exhaustion and mental deterioration arising from the prolonged and emotionally charged caregiving process. This aligns with the Stress-Burden Model, which posits that the interaction between external caregiving stressors (e.g., symptom management, financial strain) and internal appraisals (e.g., perceived responsibility, guilt) determines caregivers’ psychological outcomes [29]. In the Iranian context, caregivers’ moral obligation to persist in caregiving despite severe fatigue is reinforced by religious values emphasizing patience and altruism, further intensifying their internalized burden. Previous research supports that the intersection of emotional suppression and sociocultural expectations of devotion leads to somatic and psychological symptoms among caregivers [30–32].

The second category, Being Stuck in Caregiving Challenges, captures caregivers’ experiences of entrapment in a cycle of psychological, financial, and social stressors. The Family Systems Theory provides a conceptual framework to interpret these findings: caregiving is not an isolated activity but part of an interconnected family network [33]. As illness progresses, changes in one member’s health dynamically influence the emotional and functional balance of the entire family system. In collectivist cultures such as Iran’s, where family interdependence is highly valued, the illness of one member can generate widespread disruption of roles, communication, and cohesion [7, 34, 35]. The subtheme of “disconnection from life” underscores how caregivers may sacrifice social participation and occupational engagement to preserve the integrity of family care, reflecting both devotion and systemic imbalance.

The Neglected Caregiver category further highlights the structural absence of institutional and psychosocial support for caregivers. Despite being essential participants in the healthcare continuum, Iranian caregivers often remain invisible within the medical system. Consistent with prior studies [6, 36, 37], participants expressed dissatisfaction with the lack of empathy from medical staff and the absence of financial and psychological assistance. From a theoretical standpoint, the Stress-Burden Model and Family Systems Theory suggest that neglecting caregiver needs not only increases individual distress but also impairs the caregiving system as a whole, reducing the quality of end-of-life care. These findings indicate an urgent need to integrate family-centered support structures into Iran’s palliative care programs.

Finally, Mental Rumination on the Death of a Loved One encapsulates caregivers’ anticipatory grief, existential anxiety, and preoccupation with the impending loss. Interpreting this through the Anticipatory Grief Theory clarifies how caregivers begin mourning even before the patient’s death, oscillating between denial, acceptance, and meaning-making. This theory emphasizes that anticipatory grief can either facilitate emotional adjustment or exacerbate distress, depending on available coping resources and cultural interpretations of death [38]. Within Iranian Islamic culture, death is often framed as divine destiny, yet the emotional pain of separation remains profound. The tension between religious acceptance and human grief was evident in participants’ narratives. Thus, culturally sensitive psychoeducational interventions are essential to normalize anticipatory grief and enhance preparedness for bereavement [39–44].

Overall, while prior research has documented caregiving burdens globally, the present study extends understanding by situating these experiences within Iran’s sociocultural, familial, and spiritual context. Applying theoretical lenses such as the Stress-Burden Model, Family Systems Theory, and Anticipatory Grief Theory enriches the analytical depth of these findings and demonstrates how caregiving is simultaneously an individual struggle, a family system process, and a culturally embedded phenomenon. Future studies should further examine how these frameworks interact with cultural norms, gender roles, and healthcare structures to inform policy and practice. Moreover, emphasizing interpretive rather than descriptive reporting provides a clearer link between the findings and broader caregiving theories. Condensing repetitive details enhances readability and allows the discussion to focus on what the findings mean for caregiving practice, policy design, and research development.

## Conclusion

This study revealed that Iranian family caregivers of patients with advanced cancer experience complex, interwoven burdens encompassing physical, psychological, social, and existential dimensions. Framed within Iran’s collectivist and faith-based culture, caregiving represents both a profound act of familial devotion and a source of cumulative strain. Integrating theoretical perspectives deepens this understanding: the Stress-Burden Model highlights the interplay of external stressors and internalized expectations; the Family Systems Theory elucidates how illness reverberates through family relationships; and the Anticipatory Grief Theory explains caregivers’ emotional responses to impending loss.

These frameworks collectively underscore that caregiving challenges are not solely individual but systemic and contextual. Therefore, healthcare professionals and policymakers should design culturally sensitive interventions that recognize caregivers as “co-recipients” of care rather than peripheral participants. Practical strategies may include routine psychological screening of caregivers, structured education on end-of-life care, incorporation of family consultation in clinical decisions, and the establishment of support networks that align with cultural and spiritual values. Such initiatives could enhance caregivers’ resilience, prevent burnout, and ultimately improve the quality of both patient and family care outcomes.

### Future research directions

Future studies should examine how sociocultural factors and healthcare systems interact to shape caregiving experiences in diverse Iranian regions. Comparative research between urban and rural caregivers, or between Iranian and other Middle Eastern contexts, could clarify the role of cultural and structural determinants in caregiver burden. In addition, longitudinal or intervention-based studies are recommended to explore how targeted psychoeducational and family-centered programs can reduce caregiver distress and strengthen resilience over time.

### Limitations

This study was conducted during the COVID-19 pandemic, which limited access to hospital settings and complicated participant recruitment. Additionally, the qualitative design and small sample size restrict the generalizability of findings. Another important limitation is the cultural specificity of the data: caregivers’ experiences are shaped by Iran’s sociocultural and religious context, and therefore, interpretations may not be transferable to societies with different family structures or belief systems. Furthermore, while theoretical frameworks such as the Stress-Burden Model, Family Systems Theory, and Anticipatory Grief Theory were used retrospectively to interpret findings, future studies should prospectively integrate these frameworks during study design and data analysis to strengthen theoretical alignment. Despite these limitations, the study provides valuable insights that can inform culturally tailored interventions for family caregivers in similar settings.

## Data Availability

The raw data and supporting materials from this study are available from the corresponding author upon reasonable request.
